# Reliability of Kinovea^®^ Software and Agreement with a Three-Dimensional Motion System for Gait Analysis in Healthy Subjects

**DOI:** 10.3390/s20113154

**Published:** 2020-06-02

**Authors:** Pilar Fernández-González, Aikaterini Koutsou, Alicia Cuesta-Gómez, María Carratalá-Tejada, Juan Carlos Miangolarra-Page, Francisco Molina-Rueda

**Affiliations:** 1 International Doctorate School, Rey Juan Carlos University, 28933 Madrid, Spain; pilar.fernandez@urjc.es; 2 Motion Analysis, Ergonomics, Biomechanics and Motor Control Laboratory (LAMBECOM), Department of Physical Therapy, Occupational Therapy, Rehabilitation and Physical Medicine, Faculty of Health Sciences, Universidad Rey Juan Carlos, 28922 Madrid, Spain; aikaterini.koutsou@urjc.es (A.K.); maria.carratala@urjc.es (M.C.-T.); juan.miangolarra@urjc.es (J.C.M.-P.); francisco.molina@urjc.es (F.M.-R.); 3 Physical Medicine and Rehabilitation Service of the University Hospital of Fuenlabrada, 28942 Madrid, Spain

**Keywords:** biomechanics, agreement, gait, visual gait analysis, optical motion capture, reliability

## Abstract

Gait analysis is necessary to diagnose movement disorders. In order to reduce the costs of three-dimensional motion capture systems, new low-cost methods of motion analysis have been developed. The purpose of this study was to evaluate the inter- and intra-rater reliability of Kinovea^®^ and the agreement with a three-dimensional motion system for detecting the joint angles of the hip, knee and ankle during the initial contact phase of walking. Fifty healthy subjects participated in this study. All participants were examined twice with a one-week interval between the two appointments. The motion data were recorded using the VICON Motion System^®^ and digital video cameras. The intra-rater reliability showed a good correlation for the hip, the knee and the ankle joints (Intraclass Correlation Coefficient, ICC > 0.85) for both observers. The ICC for the inter-rater reliability was >0.90 for the hip, the knee and the ankle joints. The Bland–Altman plots showed that the magnitude of disagreement was approximately ±5° for intra-rater reliability, ±2.5° for inter-rater reliability and around ±2.5° to ±5° for Kinovea^®^ versus Vicon^®^. The ICC was good for the hip, knee and ankle angles registered with Kinovea^®^ during the initial contact of walking for both observers (intra-rater reliability) and higher for the agreement between observers (inter-rater reliability). However, the Bland–Altman plots showed disagreement between observers, measurements and systems (Kinovea^®^ vs. three-dimensional motion system) that should be considered in the interpretation of clinical evaluations.

## 1. Introduction

Gait analysis is necessary to diagnose musculoskeletal and neurological disorders, as well as evaluation of the efficacy of different interventions performed in patients [1].

For the gait evaluation, three-dimensional motion analysis systems are considered the gold standard. They provide objective and quantitative data regarding kinematic and spatiotemporal parameters [2]. However, these systems present several disadvantages, such as the high cost of equipment, the need for trained personnel, considerable processing times and the large spaces requirement for installation.

Observational scales are widely used in gait assessment. These allow us to document the state of the gait and the changes produced after the intervention, as these scales are accessible and easy to use [3,4]. In any case, the management of these tests is not exempt from the subjectivity of the evaluators.

In order to achieve more objective data than the observational scales and to reduce the costs of three-dimensional motion capture systems, new low-cost methods of motion analysis [5] have been developed in recent years. These are based on the use of videos captured by video cameras or mobile smart devices for further analysis. The most commonly used applications are: Ubersense^®^, PostureScreen^®^ or KCapture^®^ for mobile smart devices and Kinovea^®^, The Captury^®^ or SimiMotion^®^ for PC applications [5,6,7,8,9,10].

Kinovea^®^ is a free 2D motion analysis software for computers that can be used to measure kinematic parameters. This software allows to analyze video without markers although its reliability may improve with the use of passive markers [8]. Kinovea^®^ has been used by various authors to analyze running or vertical jumping in athletes [6,7,8,9,10]. Bertelsen et al. (2012) studied the inter-rater reliability of the Kinovea^®^ for the detection of the initial contact phase during running. They obtained a good correlation between them (kappa = 0.76–0.92) [6]. Later, Damsted et al. (2015) analyzed the reliability of this software, acquiring a greater intra-rater agreement (kappa = 0.83–0.88) than inter-rater agreement (kappa = 0.50–0.63) [7]. Also, Damsted et al., in another study published in 2015, investigated the reliability in the detection of hip and knee joint positions in the initial contact phase during running, obtaining a reasonable intra- and inter-rater agreement [8]. Other authors have used the Kinovea^®^ software to analyze the range of movement of the cervical spine in the sagittal plane [11] or to obtain the kinematics of the wrist joint [12]. Mathew et al. (2017) used this software to study in older adults the joint angles of ankle, knee and hip at different phases of gait cycle. The authors provide kinematic asymmetries in the participants’ gait pattern as a restriction of hip extension [13]. Therefore, the literature demonstrates the use of Kinovea^®^ software in sports and clinical settings. However, there is an absence of studies which, to our knowledge, evaluate the psychometric properties for the analysis of human gait. These circumstances explain the rationale to carry out a first study of reliability and validity.

The aims of this study were: 1) to evaluate the inter- and intra-rater reliability of Kinovea^®^ for registering the hip, knee and ankle angles during the initial contact phase of walking; 2) to study the agreement, comparing the angles obtained through Kinovea^®^ with those registered with a three-dimensional motion capture system.

## 2. Materials and Methods

### 2.1. Participants

Voluntary participation of subjects was requested through informative talks. Subjects were selected according to the following criteria: older than 18 years of age, absence of pathologies that cause alterations in gait and posture, and not using any orthosis or gait support products. Participants were excluded if they had: osteoarticular, muscular or neurological pathologies involving gait alterations or lower limb injuries within six months prior to the study.

### 2.2. Ethical Aspects

This protocol was approved by the local ethics committee (0702201703417). Informed consent was obtained from all participants included in this study.

### 2.3. Instrumentation

Gait video was recorded by a digital camera Nikon D3200 Full HD, resolution 1280 **×** 720 pixels at 50 frames per second (fps). The AF-S DS NIKKOR 18–55 mm lens at the minimum available zoom was used. It was located perpendicular to the participant at 2.5 m and 1 m above the floor.

The VICON Motion System^®^ (Oxford Metrics, Oxford, UK) was used to analyze the agreement with Kinovea^®^. This system consists of eight 100 Hz infrared cameras, three AMTI^®^ force-plates, two BASLER A601FC-2 video cameras and a data station where information is recorded and processed.

### 2.4. Procedures

The research took place at the Motion Analysis, Biomechanics, Ergonomics, and Motor Control Laboratory (LAMBECOM), located in the Physiotherapy, Occupational Therapy, Rehabilitation, and Physical Medicine Department (Faculty of Health Sciences, Rey Juan Carlos University).

**Figure 1:**
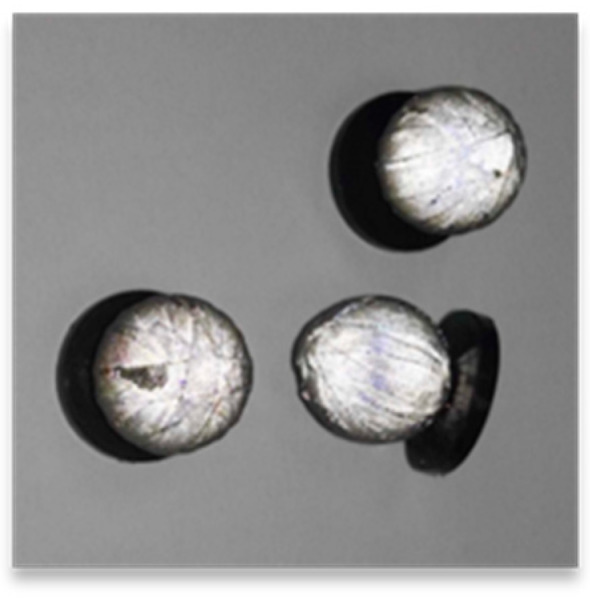
Passive and reflective markers used in this work.

All participants were evaluated twice with a separation of one week between both appointments. To carry out the movement acquisition, passive and reflective markers were placed in specific anatomical areas of the lower limbs (anterior superior iliac spine, posterior superior iliac spine, middle third of thigh, external femoral condyle, middle third of tibia, external malleolus, calcaneus and head of second metatarsal), according to the biomechanical models of Davis et al. [14] and Kadaba et al. [15]. An additional marker was placed on the greater trochanter [8,13] (Figure 1).

After the instrumentation was completed, the subjects were instructed to walk along the 11-m walkway (back and forth). They were asked to walk at a self-selected comfortable gait speed.

In order to delimit the recording area, two marks were placed on the footbridge that the subjects had to walk, at two meters between them. Two researchers were synchronized to start and stop the acquisition of motion with the digital cameras and the VICON system^®^. The recording started when the participants entered the recording area and stopped when they left it. Recordings of five repetitions per subject were made in each of the sessions.

### 2.5. Analysis of Data

The motion capture with the digital cameras and the VICON system^®^ was repeated in the first session and in the second session. One researcher distributed the videos acquired for each session between the observers and then compiled the data.

For the kinematics analysis the left lower limb was assessed. The angles of the hip, knee and ankle joints at the initial contact phase were analyzed in the sagittal plane of the studied lower limb. Kinovea^®^ version 0.8.15 was used to analyze the videos.

**Figure 2:**
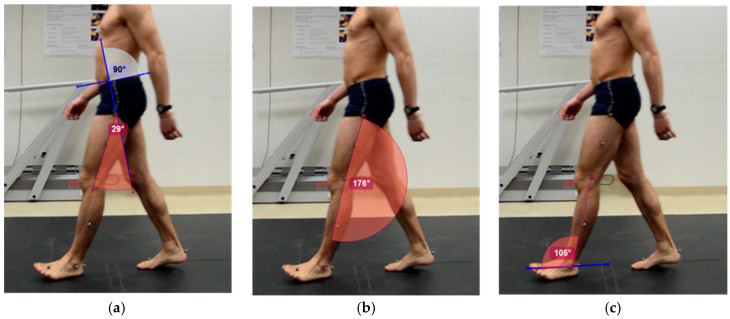
Joint angles calculation using Kinovea^®^ in (**a**) hip, (**b**) knee and (**c**) ankle.

Two observers selected the initial contact of the lower limb by observing the acquired videos, which was expected to occur at an intermediate distance between the two marks established on the footbridge. The two observers agreed to analyze the same event in the exact frame. The “angle” tool in Kinovea^®^ was used to acquire the kinematics of the hip, knee and ankle in this stage of gait. The angles calculation procedure (Figure 2) followed for each joint is presented:

- Hip. A line is drawn through the anterior superior iliac spine and the posterior superior iliac spine. Perpendicular to this, another line is drawn that passes through the greater trochanter. The angle formed by the latter and the line joining the greater trochanter to the external femoral condyle will form the joint range of the hip.
- Knee. A line is drawn between the reference points of greater trochanter and femoral condyle, and another between femoral condyle and external malleolus. The angle formed between the two lines will be used for calculating the knee joint range. In this work, 180 degrees will be considered as the neutral position of the knee. Joint range is calculated by the following equation:
  Knee Joint Range = 180-(angle obtained with Kinovea^®^), positive values correspond to knee flexion and negative values to extension.
- Ankle. A line is drawn that joins the markers of the head of the second metatarsal and the calcaneus. The angle formed between this and the line passing through the femoral condyle and the external malleolus is used to calculate the ankle joint range. In this work, 90 degrees will be considered as the neutral position of the ankle. Joint range is calculated by the following equation:
  Ankle Joint Range = 90-(angle obtained with Kinovea^®^), positive values correspond to dorsiflexion, and negative values to plantar flexion.

For the processing of trials obtained with VICON Motion System^®^ (Oxford Metrics, Oxford, UK), Vicon Nexus^®^ 1.8.5 software was used [16,17].

The initial contact with VICON was identified using a 20 N threshold on the vertical force component measured by the force plates [18]. The output angles for all joints were calculated from the YXZ cardan angles derived by comparing the relative orientations of the two segments. The course and direction of the segment axes are shown in the Vicon Plug-in Gait Product Guide [16].

**Figure 3:**
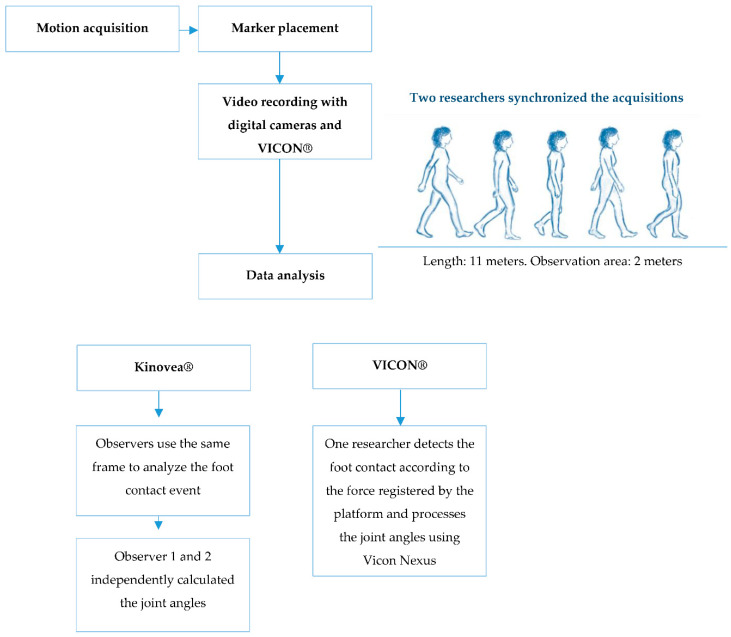
Diagram of the gait procedure and gait analysis.

Procedure and data analysis are summarized in Figure 3.

### 2.6. Sample Size Calculation

Sample size was calculated based on Walter et al. [19]. Considering a minimally acceptable Intraclass Correlation Coefficient (ICC) (p0) of 0.6, an expected ICC (p1) of 0.8, and 10% of attrition, 43 subjects are needed. Finally, the sample size consisted of 50 subjects.

### 2.7. Statistical Analysis

In order to evaluate the reliability between the two different testing sessions and between the observers, the intra-class correlation coefficient (ICC) was used [20]. The ICC was estimated, and their 95% confident intervals were calculated, using the SPSS statistical package version 22 SPSS Inc., Chicago, IL, USA), based on absolute agreement and a mixed-effect model (ICC 3,1).

Point estimates of the ICC, the r values, are interpreted as excellent (>0.9), good (0.76–0.9), moderate (0.5–0.75) and poor (lower than 0.50) [21].

Bland–Altman analysis with 95% limits of agreement was performed to assess intra and inter-rater reliability and the agreement between Kinovea^®^ and VICON^®^. The bias and the limits of agreement are shown in the plots for the parameter registered. The mean score is plotted on the *x*-axis, and the difference between observers, sessions or systems (mean of the differences) is plotted on the *y*-axis (mean of the difference ±1.96 SD, Standard Deviation). The width of the limits of agreement and the distance of the mean of the differences with respect to zero can be used to interpret the errors between measurements. Bland–Altman plots allow comparisons between two different measurement systems, observers or sessions when evaluating the same dataset to analyze the match level [22]. Dependent sample t-tests were also used to compare the mean differences between the two systems. The statistically significant *p*-value was set at 0.05.

## 3. Results

The study group consisted of 50 subjects (26 women/24 men; age 21.62 ± 2.62 years; body mass 65.74 ± 12.94 kg; height 167.49 ± 25.57 cm) without alterations in gait. There were no missing data.

**Figure 4:**
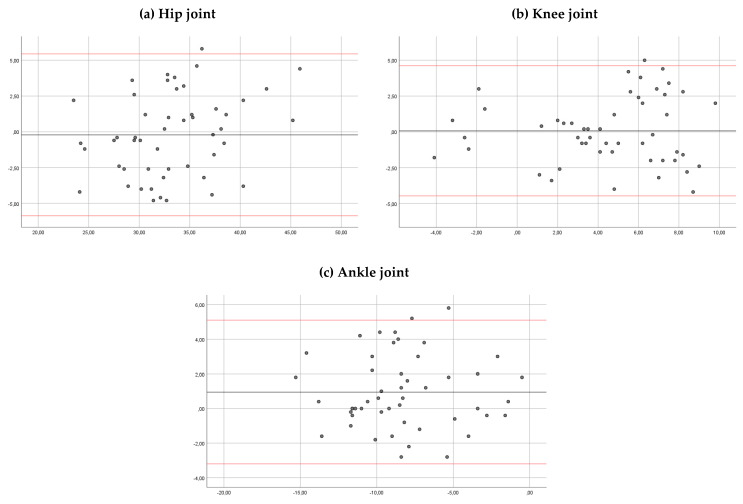
Bland–Altman plots comparing results between sessions of measurements (for observer 1) for the hip ankles (**a**), knee angles (**b**) and ankle angles (**c**). Bias (black line) and limits of agreement (red lines) are shown for each parameter. The mean score is plotted on the *x*-axis, and the difference between sessions (mean of the differences) is plotted on the *y*-axis (mean difference ± 1.96 SD).

**Figure 5:**
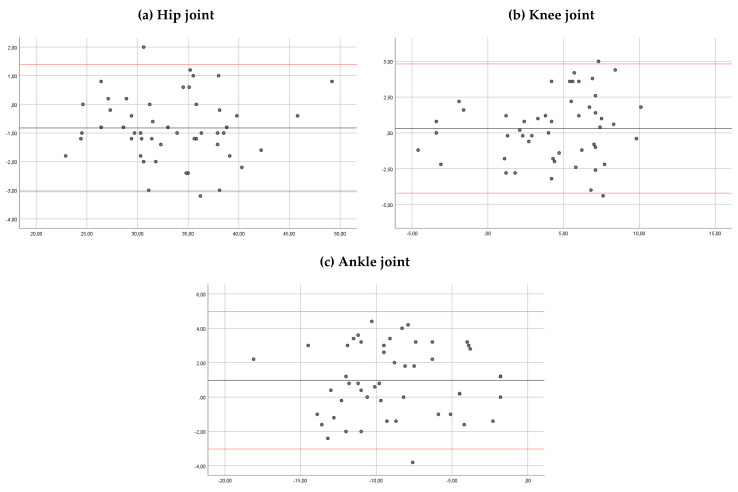
Bland–Altman plots comparing results between sessions of measurements (for observer 2) for the hip ankles (**a**), knee angles (**b**) and ankle angles (**c**). Bias (black line) and limits of agreement (red lines) are shown for each parameter. The mean score is plotted on the *x*-axis, and the difference between sessions (mean of the differences) is plotted on the *y*-axis (mean difference ± 1.96 SD).

The intra-rater reliability showed a good correlation for the hip, the knee and the ankle joints (ICC > 0.85) for both observers (Table 1). The mean of the differences between sessions for hip, knee and ankle angles were 0.21 and 0.17, 0.06 and 0.3, and 0.98 and 0.97 degrees, observer 1 and 2, respectively. In Bland–Altman plots, the limit of agreement for hip, knee and ankle angles was 5.44 to −5.87 and 6.52 to −6.17, 4.61 to −4.47 and 4.82 to −4.21, and 5.10 to −3.20 and 4.97 to −3.02 degrees, observer 1 and 2, respectively (Figure 4 and Figure 5).

**Figure 6:**
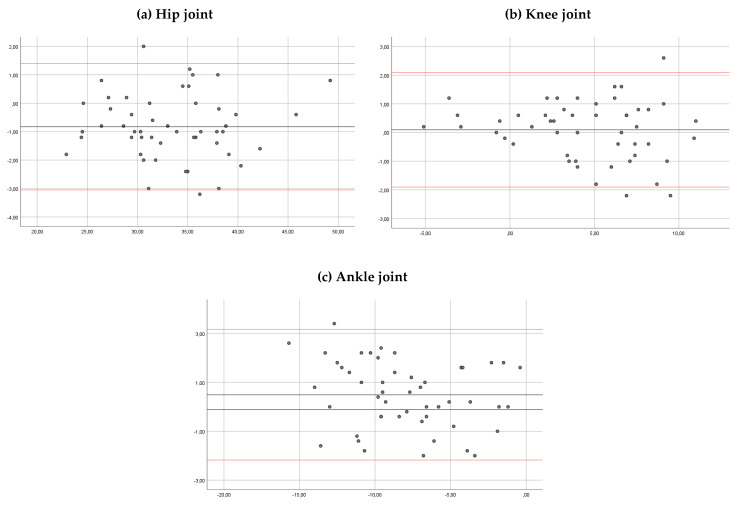
Bland–Altman plots comparing results between observers for the hip ankles (**a**), knee angles (**b**) and ankle angles (**c**). Bias (black line) and limits of agreement (red lines) are shown for each parameter. The mean score is plotted on the *x*-axis, and the difference between observers (mean of the differences) is plotted on the *y*-axis (mean difference ± 1.96 SD).

The ICC for the inter-rater reliability was >0.90 for the hip, the knee and the ankle joints in both observers (Table 2). The mean of the differences was 0.8, 0.09 and 0.48 degrees, respectively. In Bland–Altman plots, the limit of agreement for the hip, knee and ankle angles was 1.40 to −3.05, 2.09 to −1.90, and 3.16 to −2.18 degrees, respectively (Figure 6).

**Figure 7:**
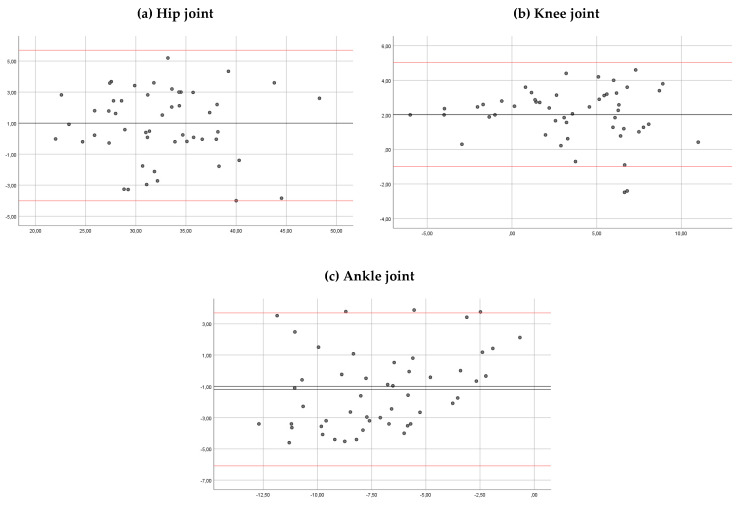
Bland–Altman plots comparing results between systems (Kinovea and Vicon) for the hip ankles (**a**), knee angles (**b**) and ankle angles (**c**). Bias (black line) and limits of agreement (red lines) are shown for each parameter. The mean score is plotted on the *x*-axis, and the difference between systems (mean of the differences) is plotted on the *y*-axis (mean difference ± 1.96 SD).

Gait parameters, measured by Kinovea^®^ and VICON^®^, are shown in Table 3. There were significant differences in the average comparison angles between two systems (*p* < 0.05). Mean differences between systems for hip, knee and ankle angles were 0.83, 2.02 and −1.19 degrees, respectively. In Bland–Altman plots, the limit of agreement for hip, knee and ankle angles was 5.26 to −3.58, 5.01 to -0.98, and 3.70 to −6.09 degrees, respectively (Figure 7).

## 4. Discussion

The purpose of the present study was to evaluate the intra- and inter-rater reliability of Kinovea^®^ and the agreement between Kinovea^®^ and VICON^®^ to obtain the joint angles during the initial contact phase of walking.

The use of systems that allow the analysis with videos, such as Kinovea^®^, could provide objective and quantitative data for advanced evaluations. Furthermore, these systems could be used not only as a diagnostic tool, but also as instruments for evaluating the results after an intervention. Its easy handling, low cost and high accessibility make it an alternative for the analysis of walking when there are no more sophisticated systems such as three-dimensional analysis equipment [1].

The main limitations of the reliability studies for Kinovea^®^ found in literature are the lack of a standardized video analysis protocol and marker placement [8,11,12,13]. Most of them used the greater trochanter as the preferred marker position [8,13]. The use of markers on the bone reliefs, as they are in the protocol presented in this work, is highly recommended as it contributes reliability to the calculation of joint ranges. This work presents discrepancies on joint angle calculations compared to studies found in literature. Damsted et al., in 2015, obtained the hip articular range considering the position of the femur with respect to the vertical [8]. According to this approach, the resulting angle would correspond to the position of the thigh ignoring the pelvis position [23]. Our approach suggests that hip angle must be calculated in relation to the pelvis.

The study of the reliability of Kinovea^®^ software is to determine that it evaluates what is intended to measure and to be able to help clinicians and researchers to interpret the data obtained by subjects with specific pathology [24]. In this sense, the ICC was good for the hip, knee and ankle angles for both observers (intra-rater reliability) and higher for the agreement between the observers (inter-rater reliability). However, the ICC has been criticized as it is a dimensionless value, therefore not easily interpreted. In this sense, Bland–Altman plots may be more useful than the ICC as they can be readily and easily interpreted in a meaningful way in both the research and clinical environment [25]. Specifically, the width of the limits of agreement are useful to understand the level of agreement or disagreement between observers, measurements or systems [26].

The Bland–Altman plots showed that for the most part the magnitude of disagreement was approximately ±5° for intra-rater reliability, ±2.5° for inter-rater reliability and around ±2.5° to ±5° for Kinovea^®^ versus Vicon^®^. In relation to the measurement errors, McGindey et al. concluded that error of 2° or less for a three-dimensional motion system is considered acceptable in a clinical situation, as such errors are probably too small to require explicit consideration during data interpretation. Errors of between 2° and 5° are also reasonable but may require consideration in data interpretation. In addition, the authors suggested that errors in excess of 5° should raise concern and may be large enough to mislead clinical interpretation [27]. Therefore, the disagreement observed in the Bland–Altman plots in this study may be reasonable for a clinical evaluation. In addition, the amplitude of the limits of agreement observed for Kinovea^®^ are similar to those obtained for a three-dimensional movement analysis system in a test–retest reliability study. Meldrum et al. found an amplitude of ± 8 degrees to detect the position of the ankle during the initial contact phase of the gait and similar results were obtained for the hip and knee kinematic parameters (±4° for ranges of motion and around ± 6° to 8° for peak kinematics in the sagittal plane) [25].

However, the results of this work should be interpreted with caution. The agreement obtained for kinovea^®^ is not enough to detect small changes between sessions and observers. Differences in joint position less than five degrees after an intervention may be due to system or observer error. There are numerous sources of variability within the testing procedure that could explain the differences between intra-rater and inter-rater reliability: marker placement error, processing errors (tester error such as in gait cycle event identification) and marker position errors [28].

Regarding the agreement between Kinovea^®^ and Vicon^®^, we found significant differences in the hip, knee and ankle angles of the systems. In addition, the Bland–Altman plots showed a disagreement between systems of ±5° for the hip and ankle angles and ±2.5° for knee angles. Lower agreement in ankle angle may be due to joint range calculation, which even taking the Vicon Plug in Gait ^®^ model [17] as a reference, still differs slightly because it defines the angle of the ankle by relating the axis of the tibia and vector of rotation of the foot (projection of the foot within the transversal plane of the laboratory). Furthermore, the camera position, which was elevated more than one meter from the ground, caused alterations in the view angle of the sagittal plane. Our results are coherent with Littrell et al. (2018) [29], who, in a technical note, analyzed the agreement of Kinovea^®^ in relation to a three-dimensional motion capture system in five subjects without pathology. They showed larger errors for the pelvis and the foot during the stance period of the gait cycle (foot = ±7.4°; pelvis = ±11.8°). These results should be considered for the clinicians when they use the Kinovea^®^ for a clinical evaluation. In addition, future studies should analyze agreement in other phases of walking and in other kinematic parameters such as joint ranges, in which there seems to be less variability [25].

### Study Limitations

The presented study has several limitations that must be pointed out. For instance, the single gait phase analysis in a single plane does not allow extrapolation of the results on reliability to the rest of gait phases and the frontal plane. However, the results would justify the start of new studies with more adequate designs.

## 5. Conclusions

The intraclass correlation coefficient was good for the hip, knee and ankle angles registered with Kinovea^®^ during the initial contact of walking for both observers (intra-rater reliability) and higher for the agreement between observers (inter-rater reliability). However, the Bland–Altman plots showed disagreement between observers, measurements and systems (Kinovea^®^ vs. three-dimensional motion system) that should be considered in the interpretation of the clinical evaluations.

## Figures and Tables

**Table 1:** Intra-rater reliability of the Kinovea parameters.

	Angles (Degrees)		Intra-Rater Reliability		
	Session 1	Session 2	ICC	95% CI	*p*
**Hip**	(a) 33.06 (5.7)	(a) 33.4 (4.87)	(a) 0.886	(a) 0.799 to 0.935	(a) < 0.01 *
	(b) 33.8 (5.52)	(b) 33.6 (5.06)	(b) 0.863	(b) 0.758 to 0.922	(b) < 0.01 *
**Knee**	(a) 4.55 (3.8)	(a) 4.36 (3.5)	(a) 0.859	(a) 0.751 to 0.920	(a) < 0.01 *
	(b) 4.45 (3.95)	(b) 4.11 (3.52)	(b) 0.868	(b) 0.768 to 0.925	(b) < 0.01 *
**Ankle**	(a) −7.77 (3.7)	(a) −8.86 (3.6)	(a) 0.875	(a) 0.780 to 0.929	(a) < 0.01 *
	(b) −8.2 (4.01)	(b) −9.46 (3.84)	(b) 0.878	(b) 0.784 to 0.931	(b) < 0.01 *

Angles are expressed in mean and standard deviation. (**a**) observer 1; (**b**) observer 2 CI, Confidence Interval. * *p*-value < 0.05.

**Table 2:** Inter-rater reliability of the Kinovea parameters.

	Angles (Degrees)		Observer 1 vs. 2		
	Observer 1	Observer 2	ICC	95% CI	*p*-Value
**Hip**	33.06 (5.7)	33.8 (5.52)	0.962	0.933 to 0.978	<0.01 *
**Knee**	4.55 (3.8)	4.45 (3.95)	0.989	0.981 to 0.994	<0.01 *
**Ankle**	−7.77 (3.7)	−8.2 (4.01)	0.973	0.952 to 0.984	<0.01 *

Kinematic are expressed in mean and standard deviation. CI, Confidence Interval. * *p*-value < 0.05.

**Table 3:** Validity of the Kinovea parameters.

	Kinematic (Degrees)		Kinovea vs. Vicon		
	Kinovea	Vicon	MD	95% CI	*p*-Value
**Hip**	33.06 (5.7)	32.2 (5.82)	0.80	0.12 to 1.49	0.022
**Knee**	4.55 (3.8)	2.53 (3.93)	2.02	1.58 to 2.45	<0.01
**Ankle**	−7.77 (3.7)	−6.50 (2.82)	−1.27	−2.03 to -0.51	<0.01

Kinematic are expressed in mean and standard deviation. MD is the mean of the differences. CI, Confidence Interval. A *p*-value < 0.05 is statistically significant.

## Abbreviations

The following abbreviations are used in this manuscript:

G.A.I.T.	Gait Assessment and Intervention Tool
LAMBECOM	Laboratory of Analysis of Movement, Biomechanics, Ergonomics and Motor Control
ICC	Intraclass Correlation Coefficient
CI	Confidence Interval
SD	Standard Deviation
MD	Mean of the differences
